# Duplicate gallbladders misdiagnosed as residual cholecystitis: A case report and review of the literature

**DOI:** 10.1097/MD.0000000000040367

**Published:** 2024-12-20

**Authors:** Xian-Shi Ma, Mei-Fa Feng, Song Ke, Liu Yang

**Affiliations:** a Department of Hepatobiliary Surgery, Yangxin People’s Hospital, Yangxin, China.

**Keywords:** duplicate gallbladder, laparoscopic cholecystectomy, MRCP, residual cholecystitis

## Abstract

**Rationale:**

Duplication of gallbladder is a rare anomaly in humans, as it is very rare for a duplication of gallbladder to be missed during the first cholecystectomy and thus require a second cholecystectomy.

**Patient concerns:**

A 42-year-old man came to our hospital with paroxysmal right upper abdominal pain for 10 days. In addition to the pain, he also had transient jaundice. He had undergone laparoscopic cholecystectomy (LC) 1 year ago. Magnetic resonance cholangiopancreatography showed gallstones, intrahepatic bile duct stones with cholangitis, no dilatation of the common bile duct, and a cystic structure was visible in the upper part of common bile duct. The patient underwent LC again, and the intraoperative diagnosis was duplication of gallbladder.

**Diagnosis:**

The patient was diagnosed with duplication of gallbladder during surgery.

**Interventions:**

The patient underwent LC again.

**Outcomes:**

The patient underwent LC again, and was diagnosed with duplicated gallbladder during the operation. The operation was successful and the patient was discharged on the 5th day after the operation without other complications.

**Lessons:**

Our case and literature review showed that there are no specific signs of duplicated gallbladders, and ultrasound has a low sensitivity for the diagnosis of double gallbladders, so double gallbladders are easily missed. Patients with double gallbladders may have multiple missed diagnoses during diagnosis and treatment, as in this case. When patients experience upper abdominal pain again after gallbladder removal, we should be alert and consider the possibility of duplicated gallbladders.

## 1. Introduction

Double gallbladder is a rare bile duct abnormality in humans, with an incidence of 1/4000. Since the incidence of gallbladder-related diseases in women is higher than that in men, the number of cases of double gallbladder is also higher than that in men.^[1–3]^ The bilobed gallbladder is classified into 3 different categories according to the commonly used Boyden classification. The first category is a bilobed gallbladder, usually in the same gallbladder fossa. The second category is the most common “H” type, with 2 separate gallbladders entering the left and right hepatic ducts or the common bile duct (CBD) through 2 different cystic ducts. The third type is the “Y” type, which enters the CBD through a single cystic duct.^[4]^ In histology and embryology, abnormal differentiation of the gallbladder may lead to the formation of multiple gallbladders when the fetus is 7 weeks old. Some researchers believe that the formation of bilobed gallbladders and “Y”-shaped double gallbladders occurs in the late development, while the formation of “H”-shaped double gallbladders occurs in the early development.^[5]^ It is worth mentioning that Flannery and Caster believed that there is another type of double gallbladder, the trabecular type, in which the accessory ducts directly discharge into the liver parenchyma, and it is difficult to observe the 2 ducts through imaging examinations.^[6]^

Most cases of duplicated gallbladders are asymptomatic and cannot be detected. Symptomatic cases of duplicated gallbladders are usually associated with gallbladder diseases such as cholecystitis or gallstones, and manifest as upper abdominal pain. If complicated by cholangitis or pancreatitis, symptoms such as jaundice, vomiting, and fever may occur.^[7,8]^ Since the clinical signs of duplicate gallbladders are the same as those of a normal single gallbladder, there are no specific signs to alert doctors. Even symptomatic duplicate gallbladders are difficult to diagnose, and imaging examinations and the patient’s medical history are often needed to assist in diagnosis. Double gallbladders are often discovered during physical examinations or in combination with other complications such as gallstones and cholecystitis. Gallstones with cholecystitis are the most common complications.^[9]^

Ultrasound (US) and magnetic resonance cholangiopancreatography (MRCP) are clinically definitive imaging modalities, and the presence of a partial double gallbladder is often diagnosed intraoperatively.^[10]^ US has always been the first choice for the examination of biliary system diseases such as cholecystitis and cholelithiasis due to its extremely high sensitivity. However, during US, the examiner can easily miss the situation of duplicated gallbladder. The surgeon may accidentally discover it during the operation, or may miss it. If it is missed during the operation, it may increase the risk of complications such as bile duct injury and even the recurrence of gallstones with cholecystitis after surgery.^[11–13]^ The accepted treatment option for symptomatic double gallbladder is surgery. For symptomatic patients, double gallbladder removal is recommended to prevent gallstones in the other gallbladder. In the past, open cholecystectomy was recommended to avoid additional intraoperative injury and to identify duplicate gallbladders.^[1,14,15]^ Nowadays, the anatomical image of double gallbladder can be displayed by three-dimensional reconstruction images obtained by MRCP before surgery. During laparoscopic cholecystectomy (LC), MRCP can provide anatomical details to avoid missing double gallbladder.^[16]^

There are many reports about LC for double gallbladders. However, because the initial doctor did not find the double gallbladder during the first cholecystectomy, the second residual cholecystectomy was performed, and a duplicate gallbladder was found during the operation. No related reports have been found before. Here, we report a case of a 42-year-old patient who did not find the second gallbladder during the first operation due to the negligence of the initial doctor. And because the surgeon was not clear about the anatomical structure of the gallbladder triangle, the gallbladder neck tube was left. This eventually led to residual cholecystitis and recurrence of gallstones with cholecystitis. We hope that this report will remind radiologists and surgeons to pay attention to the recurrence of inflammation after cholecystectomy and consider the possibility of double gallbladders, because these changes may lead to missed diagnosis, misdiagnosis, and surgical or postoperative complications.

## 2. Case report

On July 24, 2023, a 42-year-old male patient was admitted to the Department of Hepatobiliary Surgery of Yangxin County People’s Hospital in Huangshi City, Hubei Province, China. The patient complained of paroxysmal right upper abdominal pain for 10 days, negative Murphy sign, and no other special discomfort. The patient also had right upper abdominal pain 1 year ago, and computed tomography scan was performed at the local Chinese medicine hospital, which showed: multiple gallbladder stones. The doctor who first saw the patient did not read the film carefully, and recommended LC for the patient according to the routine diagnosis and treatment principles. The patient underwent LC at the local Chinese medicine hospital on April 20, 2022, and the symptoms disappeared. This time, due to mental tension during the exam, he suddenly developed upper abdominal tenderness.

**Figure 1.**
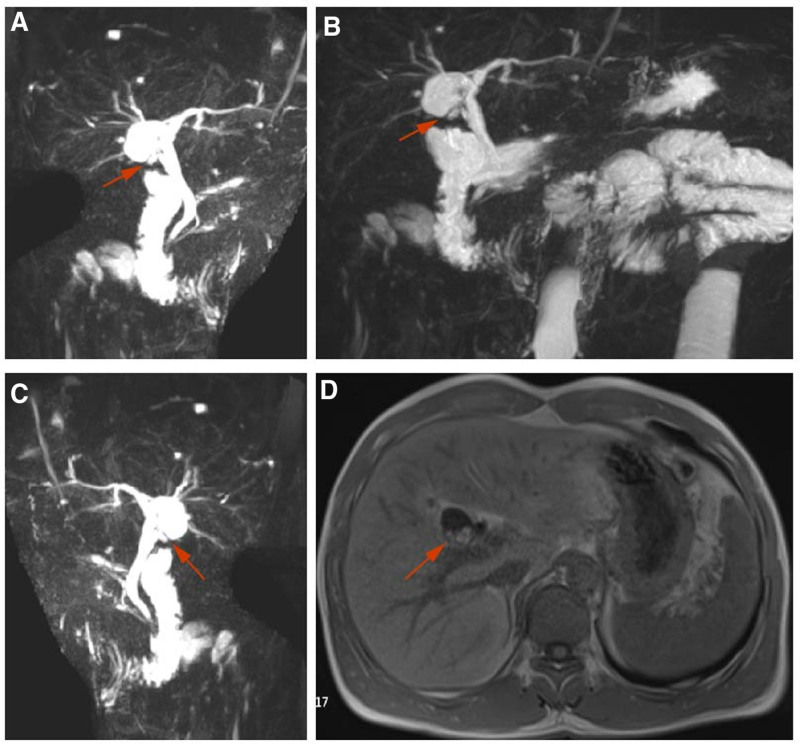
The patient’s MRCP. (A–C) Repeated gallbladder and residual cervical duct of gallbladder can be seen by MRCP radiography. (D) Repeated gallbladder stones can be seen on MRI. MRCP = magnetic resonance cholangiopancreatography.

**Figure 2.**
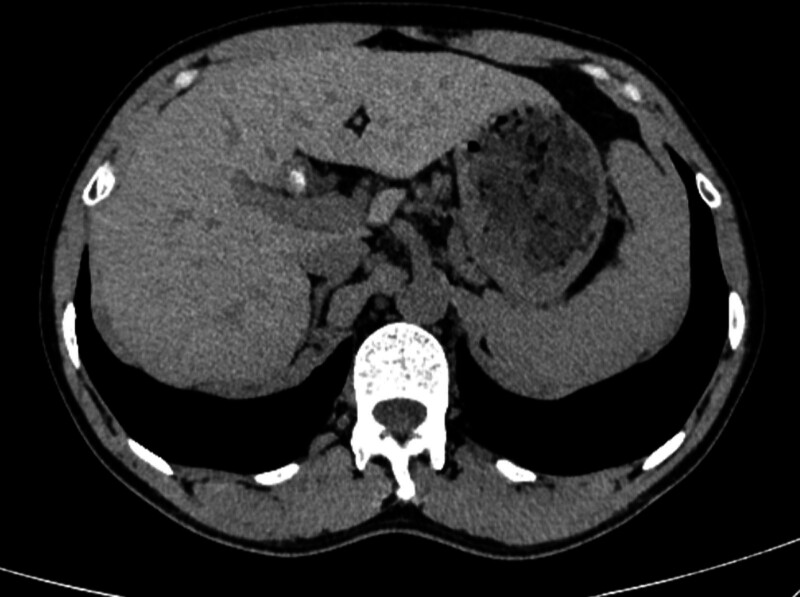
CT showed high density shadow in the repeated gallbladder. CT = computed tomography.

Come to our hospital for treatment. After asking about the medical history, we suspected bile duct stones. So MRCP was recommended, and MRCP showed: gallbladder stones, intrahepatic bile duct stones, no dilatation of the CBD, and small cysts on the top of the liver (Fig. 1). Computed tomography showed residual cholecystitis complicated by gallstones (Fig. 2). The color Doppler US of the gallbladder, spleen and pancreas showed: abnormal echoes in the gallbladder fossa area of the intrahepatic calcification, and the possibility of gallbladder stump and mud-like stones was considered high. There was no previous family history, and laboratory data showing white blood cell count (WBC: 3.5-9.5 × 10^9^/L) of 3.18 × 10^9^/L, neutrophil percentage (N%: 40–75%) of 50.0%, direct bilirubin (DBIL: 0–6.84 μmol/L) of 1.00 μmol/L, indirect bilirubin (IBIL: 0–13.7 μmol/L) of 6.30 μmol/L, alanine transaminase (ALT: 0–37 U/L) of 13 U/L, and aspartate aminotransferase (AST: 0–37 U/L) of 17 U/L. In the physical examination, there was no yellow staining of the sclera, soft abdomen, negative Murphy sign, tenderness and rebound pain (‐). Combined with the medical history and biochemical examination, we considered that it was likely to be chronic residual cholecystitis. We recommend surgical treatment.

**Figure 3.**
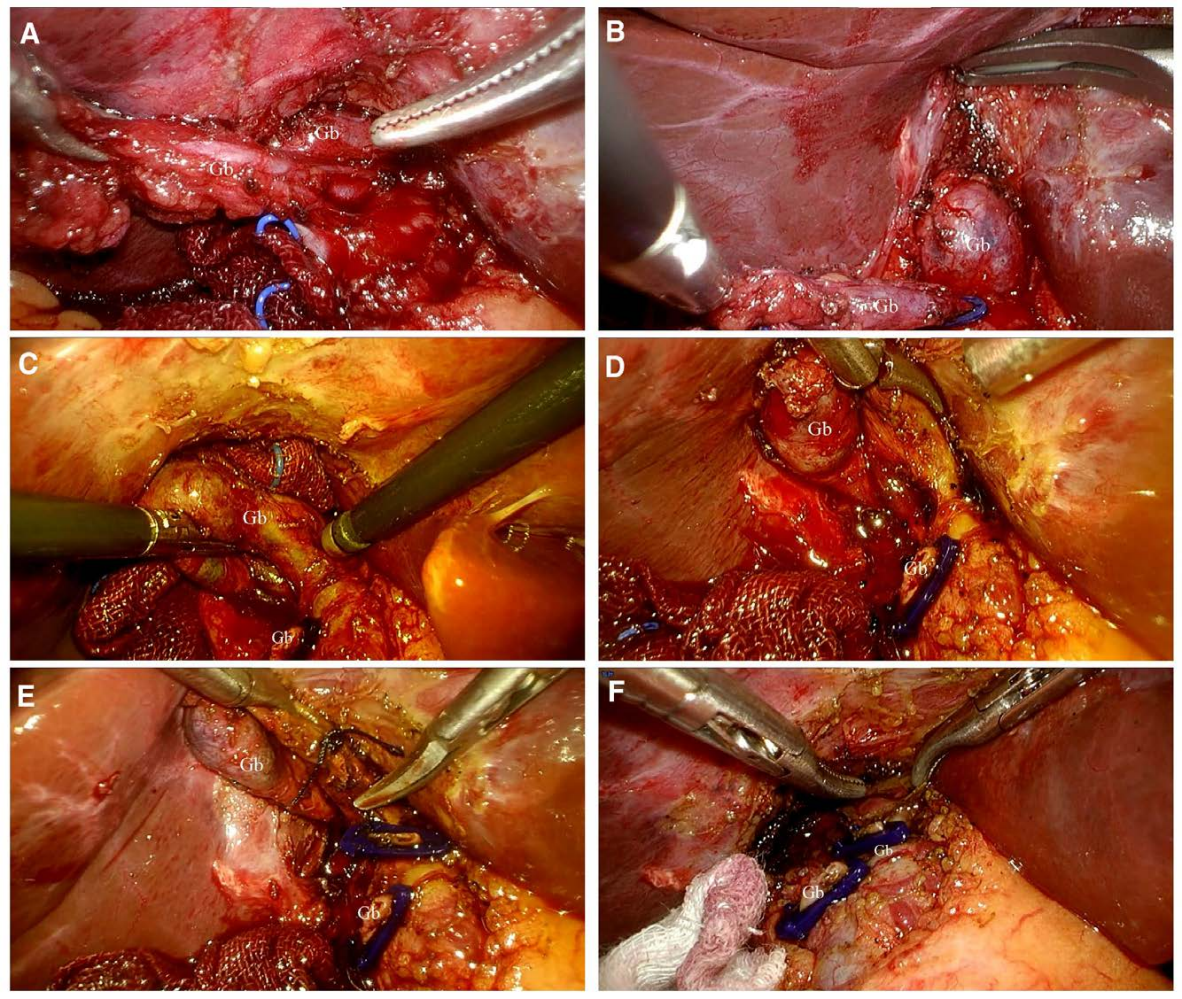
Representative photos of laparoscopic repeated cholecystectomy. (A) Dissecting the residual cervical duct of the gallbladder. (B) Exposure of repeated gallbladder. (C and D) It can be seen that there is a black sand-like stone outflow from the gallbladder. (E) Ligation and cutting of repetitive gallbladder neck duct. (F) The bottom of the repeated gallbladder is closely connected to the CBD. CBD = common bile duct.

During the operation, an incision of about 1.5 cm was made below the umbilicus, and the pneumoperitoneum needle was inserted into the abdominal cavity from the incision. After testing the negative pressure in the abdominal cavity, CO_2_ gas was injected into the abdominal cavity to form an artificial pneumoperitoneum of 13 mm Hg, using 3 trocars. Laparoscope was inserted. The patient was placed in the left lateral decubitus position with the head high and the feet low, and the whole abdominal cavity was observed. The gallbladder fossa was adhered, and the adhesion was loosened to reveal the residual gallbladder neck. The residual gallbladder neck was freed and ligated with an absorbable ligature clip. Before removing the residual gallbladder from the liver bed, a cystic object was found on the right side of the common hepatic duct, which was suspected to be a gallbladder structure and was about 3.0 * 3.0 cm in size. The film was reviewed during the operation and the MRCP was carefully read. The MRCP showed that the structure above the residual gallbladder was similar to the gallbladder, and the possibility of a second gallbladder was considered. It was decided to continue to separate the surrounding tissues, and the gallbladder was peeled off from the liver bed with difficulty, and the gallbladder body and neck were freed (Fig. 3). It was found that the bottom of the gallbladder was adhered to the wall of the common hepatic duct and could not be separated. To prevent damage to the CBD, an electric hook was used to rupture the gallbladder from the bottom of the gallbladder. Muddy black stones were seen flowing out (Video S1, Supplemental Digital Content, http://links.lww.com/MD/O189).

After the suction device was used to clean them, 2 absorbable ligatures were used on the proximal end of the gallbladder neck and the cystic artery. The clips were clamped and cut off in a conventional manner. The electric hook was placed under the serosa of the gallbladder bed until the gallbladder was completely separated from the liver. The electrocoagulation stick was used to cauterize the gallbladder bed to stop bleeding. The gallbladder was placed in a specimen bag and removed through the umbilicus. A drainage tube was placed in the gallbladder fossa and the incision was sutured. The course of the disease was regular after the operation, the drainage tube was removed, and the patient was discharged from the hospital 5 days after the operation.

## 3. Discussion

Duplication of the gallbladder is usually only discovered when there is inflammation or other disease. It is easy to miss it during US.^[17]^ Studies have shown that US has a diagnostic specificity of up to 70% for cholecystitis and gallstones, but has a low sensitivity for anatomical variations of the gallbladder, because double gallbladders, folded gallbladders, and vascular bands have similar US appearances, so it is difficult to differentiate between them, and double gallbladders can only be diagnosed occasionally.^[18–22]^ It is worth mentioning that US is the most commonly used imaging method for detecting early cholangiocarcinoma, with a sensitivity of up to 80%. Wei C et al reported a patient with cholangiocarcinoma complicated by repeated gallbladder US.^[23]^

When the patient first visited the hospital, no double gallbladder was found during preoperative examinations or during surgery. During this visit, we diagnosed the patient’s disease as residual cholecystitis combined with intrahepatic bile duct stones based on the patient’s medical history, physical signs, and MRCP results. Obviously, due to our lack of clinical experience and lack of understanding of the anatomical structure of double gallbladders, we did not diagnose double gallbladders. It might be more helpful if an MRCP was performed before the first cholecystectomy. MRCP is mainly used as the main examination method for the diagnosis of bile duct and pancreatic duct lesions. Because MRCP can directly visualize the cystic duct, is noninvasive, radiation-free, does not require anesthesia and surgery, and its accuracy rate for diagnosing cystic duct variations is as high as 95%, MRCP can provide a good preoperative examination.^[24–29]^

It is worth noting that before the operation, neither the radiologist nor the surgeon realized that this was a second gallbladder. During laparoscopic exploration, the residual cystic duct was observed under direct vision, and when the residual cystic duct was separated, a cystic structure was found on the deep surface of the caudate lobe of the liver. The surrounding adhesion tissue was separated until the gallbladder was completely exposed, and the bottom was broken by the electric hook, and muddy stones flowed out. A careful reading of the MRCP during the operation confirmed that the cystic structure was a second gallbladder, and the postoperative pathological results also confirmed this. Congenital gallbladder variations increase the risk of complications after LC, and identifying variations or abnormalities in the biliary system before reoperation can avoid surgical injuries to the bile duct to a certain extent, so preoperative examinations are very important.^[30,31]^ US has low sensitivity and great limitations. The use of MRCP can prevent serious complications of LC in such patients to a certain extent.^[32]^

This patient belongs to the “H” subtype, and double gallbladders are more likely to form CBD stones. The reason may be that the fluid dynamics in the bile duct change and form vortices. In addition, double gallbladders combined with other diseases will increase the difficulty of surgery and the probability of postoperative complications. Compared with the “Y” subtype, the “H” subtype is more likely to be converted to open surgery.^[33,34]^ Symptomatic gallbladders require surgery. Even if only one gallbladder is symptomatic, it is still recommended to remove both gallbladders. The researchers believe that this recommendation can avoid patients undergoing another cholecystectomy and the impact of recurrent cholecystitis on patients.^[24,35]^ R. Silvis et al reported a case of a patient with double gallbladder who was missed during the initial LC and was diagnosed with double gallbladder by the second laparotomy.^[13]^ Elice Borghi et al described a case of a patient who had a second gallbladder perforation after initial LC and underwent repeat LC.^[36]^ Krithika R et al reported a case of a patient who had a second attack of cholecystitis after the initial LC and was diagnosed with double gallbladder and underwent cholecystectomy.^[17]^ They demonstrated that missed double gallbladder can be successfully managed with laparoscopic reoperation, and our case also confirmed this. The disadvantage of these studies is the limited sample size, especially for patients who were diagnosed only at the second surgery, so further in-depth research is needed.

We searched for relevant articles from the PubMed database using the term “double gallbladder” and “duplication of gallbladder.” Articles published in English and involving human subjects were included. We selected all studies reporting the outcomes of double gallbladder surgery. Demographic data, surgical treatment, and postoperative outcomes were recorded and summarized. Nineteen articles met the inclusion criteria (Table S1, Supplemental Digital Content, http://links.lww.com/MD/O190). The mean age at the time of double gallbladder discovery was 41.9 years, 7 patients were H-type, 8 patients were Y-type, and 1 patient was trabecular type. MRCP was performed preoperatively in 12 patients, and 7 patients did not undergo MRCP preoperatively. Double gallbladders were found intraoperatively in 4 patients, double gallbladders were diagnosed by preoperative imaging in all 11 patients, and double gallbladders were diagnosed before the second surgery in the remaining 3 patients. Intraoperative cholangiogram was performed intraoperatively in 5 patients, and intraoperative cholangiogram was not performed intraoperatively in 14 patients. Laparoscopic was selected in all 11 patients, open surgery was selected in 2 patients, laparoscopic was converted to open surgery in 1 patient, laparoscopic was performed twice in 2 patients, and laparoscopic was performed in 1 patient for the first time and open surgery for the second time. In our case, the patient also underwent 2 laparoscopy.

## 4. Conclusion

In summary, when typical signs of cholecystitis are found and US indicates that the gallbladder may be abnormal, MRCP should be performed to effectively improve the diagnosis rate of double gallbladder. If preoperative examinations such as MRCP are not performed before surgery, intraoperative cholangiography can help identify anatomical variations of the gallbladder to reduce the risk of bile duct injury. When a patient who has undergone cholecystectomy is found to have typical signs of cholecystitis again, the attending physician should not only consider residual cholecystitis, but also the possibility of double gallbladder, and MRCP is also needed to provide sufficient basis for formulating a treatment plan.

## Author contributions

**Formal analysis:** Xian-Shi Ma.

**Visualization:** Mei-Fa Feng, Song Ke.

**Writing – original draft:** Liu Yang.

**Writing – review & editing:** Liu Yang.

## Supplementary Material


