# Supplementary material for: Duplicate gallbladders misdiagnosed as residual cholecystitis: A case report and review of the literature
Source: Medicine (Baltimore). 2024 Dec 20;103(51):e40367. doi: 10.1097/MD.0000000000040367 (PMC11666152; doi:10.1097/MD.0000000000040367)
Supplement: Supplementary file 2 [file medi-103-e40367-s002.docx]

**Supplemental Table1**

| **Author** | **Age** | **Sex** | **Duplication type** | **Preoperative MRCP** | **Diagnosis** | **IOC** | **Operative approach** |
| --- | --- | --- | --- | --- | --- | --- | --- |
| Abdullah O[7] | 4 | F | Y | No | Preoperative US | No | Laparoscopic |
| Süreyya BG[28] | 10 | M | Y | Yes | Preoperative US | No | Laparoscopic |
| Semeret M[4] | 34 | F | Trabecular type | No | Intraoperative | Yes | Laparoscopic converted to open |
| Abdulaziz AA[8] | 26 | F | Y | Yes | Preoperative US | No | Open |
| Wei C[24] | 58 | F | H | Yes | Preoperative US | No | NA |
| WEI Y[33] | 66 | F | H | Yes | Preoperative US | No | Laparoscopic |
| Abdulwahid MS[6] | 33 | F | Y | No | Intraoperative | No | Laparoscopic |
| D. Urbain[30] | 19 | M | NA | No | Preoperative ERCP | Yes | No mentioned |
| Duminda S[34] | 41 | M | H | No | Intraoperative | No | Laparoscopic |
| Wei S P[31] | 76 | M | Y | Yes | Intraoperative | Yes | Laparoscopic |
| Hongkai Z[35] | 61 | M | H | Yes | Intraoperative | No | Open |
| Mohammed SA[5] | 43 | F | Y | No | Intraoperative | No | Laparoscopic |
| Mohammud G[14] | 39 | F | Y | Yes | Preoperative MRCP, CT | No | Laparoscopic |
| Mary K[15] | 17 | F | H | Yes | Preoperative MRCP, CT | Yes | Laparoscopic |
| JLC Smelt[22] | 75 | F | H | Yes | Preoperative MRCP | No | Laparoscopic |
| Sumanta K G[29] | 21 | M | Y | Yes | Preoperative MRCP | Yes | Laparoscopic |
| Felice B[36] | 72 | M | NA | No | Second preoperative CT | No | Two laparoscopy |
| Krithika R[17] | 46 | M | H | Yes | Second preoperative US | No | Two laparoscopy |
| R. Silvis[13] | 56 | F | NA | Yes | Second preoperative US, CT | No | First laparoscopic, second open |
